# Effects of miR-33a-5P on ABCA1/G1-Mediated Cholesterol Efflux under Inflammatory Stress in THP-1 Macrophages

**DOI:** 10.1371/journal.pone.0109722

**Published:** 2014-10-17

**Authors:** Min Mao, Han Lei, Qing Liu, Yaxi Chen, Lei Zhao, Qing Li, Suxin Luo, Zhong Zuo, Quan He, Wei Huang, Nan Zhang, Chao Zhou, Xiong Z. Ruan

**Affiliations:** 1 Department of Cardiology, The First Affiliated Hospital of Chongqing Medical University, Chongqing, P.R. China; 2 Centre for Lipid Research, Key Laboratory of Metabolism on Lipid and Glucose, Chongqing Medical University, Chongqing, P.R. China; 3 Centre for Clinical Research, The First Affiliated Hospital of Chongqing Medical University, Chongqing, P.R. China; 4 John Moorhead Research Laboratory, Centre for Nephrology, University College London Medical School, Royal Free Campus, London, United Kingdom; University of Padova, Italy

## Abstract

The present study is to investigate whether inflammatory cytokines inhibit ABCA1/ABCG1-mediated cholesterol efflux by regulating miR-33a-5P in THP-1 macrophages. We used interleukin-6 and tumor necrosis factor-alpha in the presence or absence of native low density lipoprotein (LDL) to stimulate THP-1 macrophages. THP-1 macrophages were infected by either control lentivirus vectors or lentivirus encoding miR-33a-5P or antisense miR-33a-5P. The effects of inflammatory cytokines, miR-33a-5P and antisense miR-33a-5P on intracellular lipids accumulation and intracellular cholesterol contents were assessed by oil red O staining and quantitative intracellular cholesterol assay. ApoA-I-mediated cholesterol efflux was examined using the fluorescent sterol (BODIPY-cholesterol). The gene and protein expressions of the molecules involved in cholesterol trafficking were examined using quantitative real-time polymerase chain reaction and Western blotting. Inflammatory cytokines or miR-33a-5P increased intracellular lipid accumulation and decreased apoA-I-mediated cholesterol efflux via decreasing the expression of ABCA1 and ABCG1 in the absence or presence of LDL in THP-1 macrophages. However, antisense miR-33a-5P reversed the effects of inflammatory cytokines on intracellular lipid accumulation, cholesterol efflux, and the expression of miR-33a-5P, ABCA1 and ABCG1 in the absence or presence of LDL in THP-1 macrophages. This study indicated that inflammatory cytokines inhibited ABCA1/ABCG1-mediated cholesterol efflux by up-regulating miR-33a-5P in THP-1 macrophages.

## Introduction

Atherosclerosis is a complex disease in which the artery wall becomes thicker and thicker due to the accumulation of plaques along the walls that finally blocks blood flow. Atherosclerosis can cause several conditions like aneurysm and rupturing of the arteries which lead to serious health problems, including heart attack, stroke and peripheral vascular disease, etc. These problems are the most common causes of worldwide morbidity and mortality [1]. Atherosclerosis involves multiple risk factors. The excessive cholesterol accumulation and inflammation in vessel walls are key factors for the development of atherosclerosis [2]. Under the effects of many risk factors, especially the long-term effects of inflammation, macrophages that derive from mononuclear cells and vascular smooth muscle cells in blood vessel walls swallow a large number of lipoprotein particles, and become foam cells. Many types of inflammatory cytokines secreted by numerous foam cells promote the intake of lipids by macrophages and vascular smooth muscle cells, leading to lesion formation and complications such as plaque disruption [3], [4]. Inflammation can facilitate arterial hyperplasia and lipid accumulation, and regulate aspects of plaque biology that triggers thrombotic complications of atherosclerosis, even in the absence of traditional risk factors. Inflammation provides a pathway that links alterations in traditional risk factors and modifications in the biology of the artery walls, leading to atherosclerosis and its complications [5], [6].

Intracellular cholesterol homeostasis is maintained by intracellular cholesterol biosynthesis, extracellular cholesterol intake and intracellular cholesterol efflux. When cells are depleted of cholesterol, sterol-regulatory element-binding protein 2 (SREBP2) is activated, promoting the synthesis of intracellular cholesterol and cholesterol intake. When intracellular cholesterol is overloaded, the expression of SREBP2 is decreased, leading to the decrease of cholesterol uptake and de novo synthesis. Meanwhile, the expression of adenosine triphosphate (ATP)-binding membrane cassette transporters A1 (ABCA1) and G1 (ABCG1) is up-regulated, resulting in the increase of cholesterol efflux [7], [8]. Cholesterol efflux impairment in macrophages will affect the progress of atherosclerosis [9]. Cholesterol efflux is the key step in cholesterol reverse transportation. ABCA1 and ABCG1 are responsible for the major part of macrophage cholesterol efflux to serum or to high-density lipoprotein (HDL) in macrophage foam cells. ABCA1 mediates the efflux of cholesterol and phospholipid from macrophages to apolipoprotein with poor lipid molecule like apolipoprotein A-I (apoA-I) [8], [10], [11]. ABCG1 mediates cholesterol efflux from macrophages to apolipoprotein with large lipid molecule like HDL [12]–[14].

MicroRNAs (miRNAs) are small, non-coding RNAs with 20–24 nucleotides that promote the down-regulation of their target genes by binding to complementary target sites in the 3′ untranslated regions (3′UTRs) of the target mRNA. miRNAs can be transcribed from their own promoters or encoded in the introns of other genes. It is speculated that in the latter case, these miRNAs might be expressed when the “hosting” mRNA is transcribed. A single miRNA can have multiple targets, potentially providing simultaneous regulation of the genes involved in a physiological pathway [15]. Recent studies showed that miR-33 is closely related to lipid metabolism [16]–[19]. Two isoforms of miR-33 (miR-33a and miR-33b) exist in human beings. miR-33a is located in intron 16 of the SREBP-2 gene (on chromosome 22) that is involved in cholesterol biosynthesis and cholesterol uptake, and is co-transcribed with SREBP-2. miR-33a binds to the 3′UTR of ABCA1/ABCG1 mRNA to inhibit ABCA1/ABCG1 translation and cholesterol efflux [20]–[24]. Mature miR-33a produces two single-stranded miRNAs (miR-33a-5P and miR-33a-3P). However, only miR-33a-5P can bind to the 3′UTR of ABCA1/ABCG1 mRNA to inhibit their translation. miR-33b is presented in intron 17 of the SREBP-1 gene (on chromosome 17) that is involved in fatty acid and triglyceride synthesis [15], [25], [26].

We have previously demonstrated that inflammation increases cholesterol accumulation by disrupting SREBP2-LDLR negative feedback regulation. Inflammatory cytokines can promote the engulfment of a large number of low-density lipoprotein (LDL) in THP-1 macrophages, vascular smooth muscle cells, liver primary cells, and HepG2 (human hepatoblastoma) cells, leading to the gathering of plenty of cholesterol in these cells [27]–[34]. We also demonstrated that inflammation can inhibit the expression of ABCA1/ABCG1, and decrease ABCA1/ABCG1-mediated cholesterol efflux. However, the mechanism by which inflammation inhibits cholesterol efflux remains unclear [32], [35]. In this study, we investigate whether inflammation activates miR-33a transcription, which inhibits ABCA1/ABCG1 gene expression and cholesterol efflux in human THP-1 macrophages.

## Materials and Methods

### Cells culture

Human monocyte cell line (THP-1) was obtained from the American Type Culture Collection (ATCC, No. TIB-202). THP-1 was cultured in RPMI 1640 medium containing 10% (V/V) fetal calf serum, 2 mmol/l glutamine, 100 U/ml penicillin, and 100 mg/ml streptomycin. THP-1 was fully differentiated into macrophages after being triggered with 160 nmol/l phorbol-12-myristate-13-acetate (PMA) for 72 h, and the differentiated THP-1 macrophages were washed extensively with phosphate-buffered saline (PBS) before use. Experiments were performed in serum-free experimental medium containing RPMI 1640, 0.2% (W/V) bovine serum albumin (BSA), 2 mmol/l glutamine, 100 U/ml penicillin, 100 mg/ml streptomycin with the anti-oxidants EDTA and butylated hydroxytoluene (BHT) at final concentrations of 100 mmol/l and 20 mmol/l, respectively (Sigma-Aldrich, St. Louis, MO, USA). All reagents for cell culture were obtained from Hyclone (Beijing, China). BSA and PMA were obtained from Sigma-Aldrich (USA). Recombinant human interleukin (IL)-6 and tumor necrosis factor (TNF)-α were obtained from SinoBio (Shanghai, China) and PeproTech Asia (USA), respectively.

### LDL preparation

LDL was isolated from fresh plasma of healthy human volunteers by sequential density gradient ultracentrifugation as described previously [28]. LDL protein concentration was determined by Lowry assay [36]. Prior written and informed consents were obtained from all healthy human volunteers. The study was approved by the Research and Development (R&D) Committee of Chongqing Medical University. Our study protocols adhered to the tenets of the Declaration of Helsinki for experiments involving human samples.

### Lentivirus infection

Lentivirus vectors encoding miR-33a-5P and anti-miR-33a-5P were constructed and transfected into the cells. Two lentivirus vectors, GV259 (Con-miR) and GV159 (Con-anti-miR), were used as controls for miR-33a and anti-miR-33a, respectively. All lentivirus were obtained from Shanghai Ji Kai Gene Chemical Technology Co., LTD. THP-1 macrophages were infected using the lentivirus after 24 h PMA stimulation according to the manufacturer’s protocol. The medium containing lentivirus and polybrene (5 µg/ml) was well mixed by gently flapping culture plate in horizontal direction. The culture medium containing 160 nmol/l PMA was replaced after 48 h of infection and the percentage of fluorescence positive cells (>80%) was examined by checking the expression of green fluorescent protein after 96 h of infection.

### Morphological examination

THP-1 macrophages with or without lentivirus infection were incubated in chamber slides in serum-free experimental medium or experimental medium with 40 ng/ml IL-6, 50 ng/ml TNF-α, 25 µg/ml LDL, 40 ng/ml IL-6 plus 25 µg/ml LDL, or 50 ng/ml TNF-α plus 25 µg/ml LDL. After 24 h incubation, the cells were washed three times in PBS, fixed for 30 min with 5% formalin solution in PBS, stained with oil red O for 30 min, and counterstained with hematoxylin for another 5 min. Finally, the cells were examined by light microscopy (Axio Imager 2, Zeiss, Germany). Six randomly selected high-power fields (HPF) (400×) were selected for examination. Semi-quantitative analysis of oil red O positive staining was performed by the Image-J software.

### Quantitative measurement of intracellular cholesterol

THP-1 macrophages with or without lentivirus infection were cultured in serum-free experimental medium in 6-well plates as previously mentioned for 24 h. Cells were then washed three times with PBS. Intracellular lipids were extracted by chloroform/methanol (2∶1) mixture and dried under vacuum, and the total cholesterol (TC) and free cholesterol (FC) contents were measured by an enzymatic assay normalized by total cell proteins determined by Lowry assay. The concentration of cholesterol ester (CE) was calculated from TC and FC.

### Cholesterol efflux

We examined apoA-I-mediated cholesterol efflux using fluorescent sterol (boron dipyrromethene difluoride linked to sterol carbon-24, BODIPY-cholesterol) [37], [38]. THP-1 macrophages with or without lentivirus infection were cultured in 96-well plates, and then cultured in serum-free medium containing 0.1 ml labeling media for 1 h. Labeling medium contains BODIPY-cholesterol (Avanti Polar Lipids), methyl-β-cyclodextrin (Sigma-Aldrich, USA), MEM-HEPES (Sangon, China), and egg phosphatidylcholine (Avanti Polar Lipids, USA). The final concentrations of BODIPY-cholesterol, egg phosphatidylcholine, and methyl-β-cyclodextrin in the labeling medium were 0.025 mM, 0.1 mM, and 10 mM, respectively. The cells were washed twice with MEM-HEPES, and then cultured in serum-free medium containing treatment factors for 18 h. Next, the cells were cultured in serum-free medium containing treatment factors and 10 µg/ml apoA-I (Sigma-Aldrich, USA) for 4 h. Single layer cells were dissolved in 0.1 N NaOH overnight. After centrifugation at 10,000 rpm for 10 min, the liquid supernatant was collected and the fluorescence intensity value that represented total cholesterol efflux was recorded using BioTek microplate reader (excitation 482 nm, emission 515 nm). The fluorescence intensity value of liquid supernatant of cells cultured in serum-free medium without treatment factor and 10 µg/ml apoA-I represented background cholesterol efflux. The apoA-I-mediated cholesterol efflux is that the balance between the total cholesterol efflux and background cholesterol efflux. The rate of apoA-I-mediated cholesterol efflux = apoA-I-mediated cholesterol efflux/(intracellular cholesterol + total cholesterol efflux)×100%.

### Quantitative real-time polymerase chain reaction (RT-PCR)

Total RNA was isolated from cultured THP-1 macrophages using RNAiso kit (Takara, Dalian, China) according to the manufacturer’s protocol. Total RNA (1 mg) was used as the template for reverse transcription using a PrimeScriptH RT reagent Kit (Takara, Dalian, China). RT-PCR was performed by a Sequence Detection System (CFX96™ Real-Time PCR Detection System, Bio-Rad, USA) using Power SYBR Green PCR master mix (Takara, Dalian, China). U6 and β-actin served as the reference housekeeping genes. U6 and miR-33a-5P were analyzed by Bulge-Loop miRNA RT-PCR. The primers of U6 and miR-33a-5P were purchased from Guangzhou RiboBio Co., Ltd. (miRQ0000091-1-2, MQP-0202). The 2^−ΔΔCt^ method was applied to obtain the relative levels of target genes. The amplification efficiencies of the target and reference were shown to be approximately 95–100%. Controls (H_2_O or samples) that were not reversely transcribed were negative for target and reference. The primer sequences used in this study were shown in Table 1.

**Table 1:** Primer sequences for RT-PCR.

Primers	Sequences
SREBP2 forward	5′-CCGCCTGTTCCGATGTACAC-3′
SREBP2 reverse	5′-TGCACATTCAGCCAGGTTCA-3′
ABCA1 forward	5′-TCCAGGCCAGTACGGAATTC-3′
ABCA1 reverse	5′-ACTTTCCTCGCCAAACCAGTAG-3′
ABCG1 forward	5′-CCCTCAGAATGCCAGCAGTT-3′
ABCG1 reverse	5′-CCGAGACACACACCGACTTG-3′
β-actin forward	5′-CCTGGCACCCAGCACAAT-3′
β-actin reverse	5′-GCCGATCCACACGGAGTA-3′

### Western blotting

Identical amounts of protein from cultured THP-1 macrophage extracts or nuclear extracts was denatured and then subjected to electrophoresis on 6% sodium dodecyl sulfate or 8% sodium dodecyl sulfate-polyacrylamide gel in a Bio-Rad mini protein apparatus (Bio-Rad Laboratories, UK). Electrophoretic transfer to nitrocellulose was accomplished at 85 V, 220 mA for 3 h. The membrane was then blocked with 5% skimmed milk for 1–2 h at room temperature and probed with rabbit anti-human SREBP2 polyclonal antibody (1∶500 dilution; Santa Cruz Biotechnology, Wiltshire, UK), rabbit anti-ATP-binding cassette transporter A1 (ABCA1) antibody (1∶1000 dilution; Abcam, USA), rabbit anti-ATP-binding cassette transporter G1 (ABCG1) antibody (1∶1000 dilution; Abcam, USA), and mouse anti-human β-actin polyclonal antibody (1∶2000 dilution; Abcam, USA) in antibody dilution buffer (5% skimmed milk in phosphate buffered saline containing 1% Tween) at 4°C overnight. Followed by three 5-min washes in PBST, they were further incubated with horseradish peroxidase-conjugated goat anti-rabbit IgG or goat anti-mouse IgG (1∶4000 dilution; ZSGB-BIO, Beijing, China) at room temperature for another 1 h. After washing, the membranes were subjected to enhanced chemiluminescence advanced system (Amersham Biosciences, USA) to be exposed. β-actin was used as loading control. The protein bands on the membrane were analyzed by a biological image analysis system to assess the protein levels.

### Statistical analysis

SPSS software version 19.0 was applied to perform statistical analysis with analysis of variance. In all experiments, data were presented as means ± SD. Pairwise comparisons (LSD test) or Dunnett’s T3 between groups were carried out. P<0.05 was considered statistically significant.

## Results

### Inflammatory cytokines increase intracellular lipid accumulation and decrease apoA-I-mediated cholesterol efflux in THP-1 macrophages

**Figure 1:**
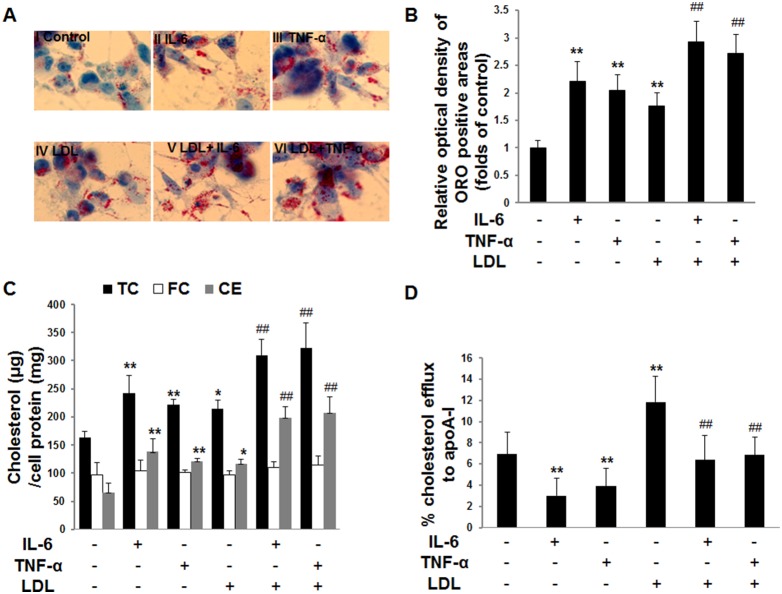
Effects of inflammatory cytokines on intracellular lipids accumulation and intracellular cholesterol efflux in THP-1 macrophages. THP-1 macrophages were incubated in serum-free medium at 37°C for 24 h. The medium was then replaced by fresh serum-free medium containing **(I)** blank control, **(II)** 40 ng/ml IL-6, **(III)** 50 ng/ml TNF-α, **(IV)** 25 µg/ml LDL, **(V)** 25 µg/ml LDL plus 40 ng/ml IL-6, or **(VI)** 25 µg/ml LDL plus 50 ng/ml TNF-α, followed by incubation at 37°C for 24 h. (**A**) The cells stained with oil red O for the examination of lipid inclusions. The results are representative of those observed in six separate experiments (×400). (**B**) Semi-quantitative analysis of oil red O positive staining was performed by the Image-J software. Data are means ± SD from 6 separate fields. (**C**) Intracellular total cholesterol (TC), free cholesterol (FC) and cholesterol ester (CE). Values are means ± SD of duplicate wells from 6 experiments. (**D**) Intracellular apoA-I-mediated cholesterol efflux. Data are means ± SD of duplicate wells from 6 experiments. *, P<0.05 compared with control; **, P<0.01 compared with control; #, P<0.05 compared with LDL; ##, P<0.01 compared with LDL.

To investigate intracellular lipid accumulation in response to inflammatory cytokines in THP-1 macrophages, we measured intracellular cholesterol levels in THP-1 macrophages. Both inflammatory cytokines IL-6 and TNF-α increased lipid accumulation as evidenced by oil red O staining (Fig. 1A) and semi-quantitative analysis (Fig. 1B) in the absence (Fig. 1A: II or III vs I) or presence (Fig. 1A: V or VI vs IV) of LDL in THP-1 macrophages. Total cholesterol (TC) and cholesterol ester (CE) were increased in inflammatory cytokine-treated cells in the absence or presence of LDL, while free cholesterol (FC) was unchanged (Fig. 1C). Meanwhile, apoA-I-mediated cholesterol efflux was decreased in inflammatory cytokine-treated cells in the absence or presence of LDL (Fig. 1D). These results suggested that inflammatory cytokines increased intracellular lipid accumulation due to decreased apoA-I-mediated cholesterol efflux in THP-1 macrophages.

### Inflammatory cytokines up-regulate the expression of miR-33a-5P and SREBP2, and down-regulate the expression of ABCA1 and ABCG1

**Figure 2:**
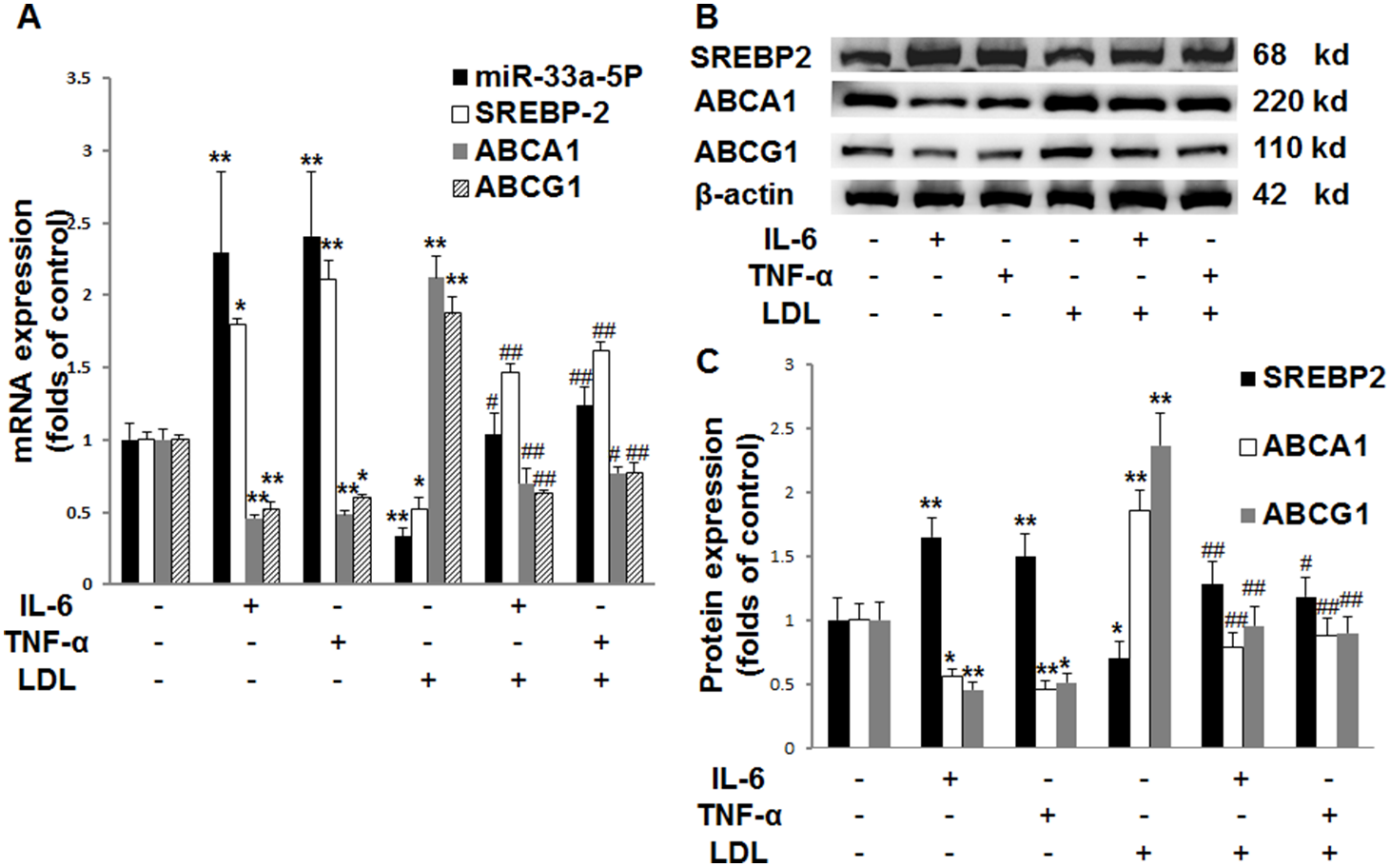
Effects of inflammatory cytokines on miR-33a-5P, SREBP2, ABCA1 and ABCG1 expression in THP-1 macrophages. THP-1 macrophages were incubated in serum-free medium at 37°C for 24 h. The medium was then replaced by fresh serum-free medium containing blank control, 40 ng/ml IL-6, 50 ng/ml TNF-α, 25 µg/ml LDL, 25 µg/ml LDL plus 40 ng/ml IL-6, or 25 µg/ml LDL plus 50 ng/ml TNF-α, followed by incubation at 37°C for 24 h. (**A**) mRNA levels of miR-33a-5P, SREBP2, ABCA1 and ABCG1 in THP-1 macrophages. The mRNA levels were determined using the 2^−ΔΔCt^ method for RT-PCR as described in the Materials and Methods section. U6 or β-actin served as a reference gene. Data are means ± SD from 6 experiments. (**B**) SREBP2, ABCA1 and ABCG1 protein levels examined by Western blotting assay. (**C**) Quantification of SREBP2, ABCA1 and ABCG1 protein levels. Histograms represent the densitometric values of SREBP2, ABCA1 and ABCG1 protein bands from four experiments, normalized to β-actin and expressed as a percentage of control. Data are means ± SD. *, P<0.05 compared with control; **, P<0.01 compared with control; #, P<0.05 compared with LDL; ##, P<0.01 compared with LDL.

Either IL-6 or TNF-α increased miR-33a-5P expression, up-regulated both mRNA (Fig. 2A) and protein (Fig. 2B and C) expression of SREBP2, and down-regulated both mRNA (Fig. 2A) and protein (Fig. 2B and C) expression of ABCA1 and ABCG1 in the absence or presence of LDL. The presence of LDL inhibited the expression of miR-33a-5P and SREBP2, but enhanced the expression of ABCA1 and ABCG1 (Fig. 2A–C). However, inflammatory cytokines overrode the effects of LDL on these molecules (Fig. 2A–C). These results suggested that inflammatory cytokines up-regulated the expression of miR-33a-5P and SREBP2, and down-regulated the expression of ABCA1 and ABCG1 in the absence or presence of LDL.

### Effects of Con-miR and Con-Anti-miR on intracellular lipid accumulation, cholesterol efflux and the expression of miR-33a-5P, SREBP2, ABCA1 and ABCG1

**Figure 3:**
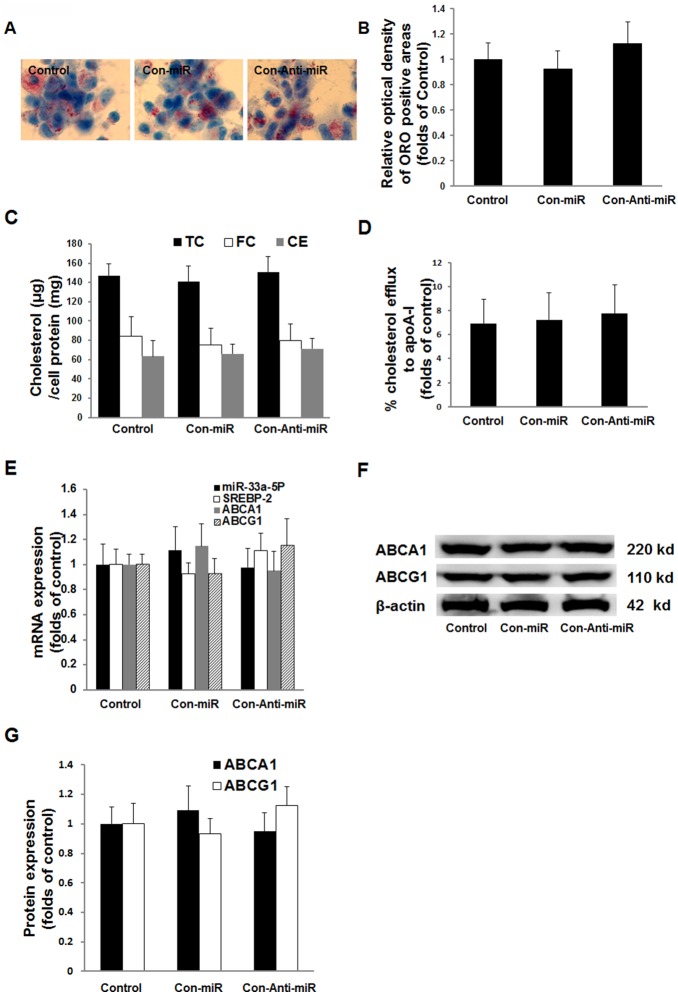
Effects of Con-miR and Con-Anti-miR on intracellular cholesterol accumulation, cholesterol efflux and the expression of miR-33a-5P, SREBP2, ABCA1 and ABCG1 in THP-1 macrophages. THP-1 macrophages were incubated in serum-free medium at 37°C for 24 h, after lentivirus (Con-miR and Con-Anti-miR) infection for 48 h. (**A**) THP-1 macrophages examined for lipid inclusions by oil red O staining. (**B**) Semi-quantitative analysis of oil red O positive staining of THP-1 macrophages for the examination of lipid inclusions. Data are means ± SD from 6 separate fields. (**C**) Intracellular free cholesterol (FC), total cholesterol (TC) and cholesterol ester (CE). (**D**) ApoA-I-mediated cholesterol efflux. Data are means ± SD of duplicate wells from 6 experiments. (**E**) mRNA levels determined by the 2^−ΔΔCt^ method for RT-PCR. U6 or β-actin served as a reference gene. Data are means ± SD from 6 experiments. (**F**) Protein levels examined by Western blotting. (**G**) Quantification of the densitometric values of SREBP2, ABCA1 and ABCG1 protein bands from four experiments, normalized to β-actin and expressed as a percentage of the control. Data are means ± SD.

The data showed that the level of intracellular cholesterol accumulation in THP-1 macrophages infected by Con-miR and Con-Anti-miR was not different from that in the uninfected control (Fig. 3A and B). Similarly, the levels of intracellular total cholesterol (TC), free cholesterol (FC), cholesterol ester (CE) in THP-1 macrophages infected by Con-miR and Con-Anti-miR were not different from that in the uninfected control (Fig. 3C). In addition, apoA-I-mediated cholesterol efflux (Fig. 3D) and the expression of miR-33a-5P, SREBP2, ABCA1 and ABCG1 (Fig. 3E, F and G) in THP-1 macrophages infected by Con-miR and Con-Anti-miR were not different from that in the uninfected control. These results indicated that Con-miR and Con-Anti-miR did not affect intracellular lipids accumulation, cholesterol efflux and the expression of miR-33a-5P, SREBP2, ABCA1 and ABCG1.

### Effects of overexpression of miR-33a-5P and anti-miR-33a-5P on intracellular lipid accumulation

**Figure 4:**
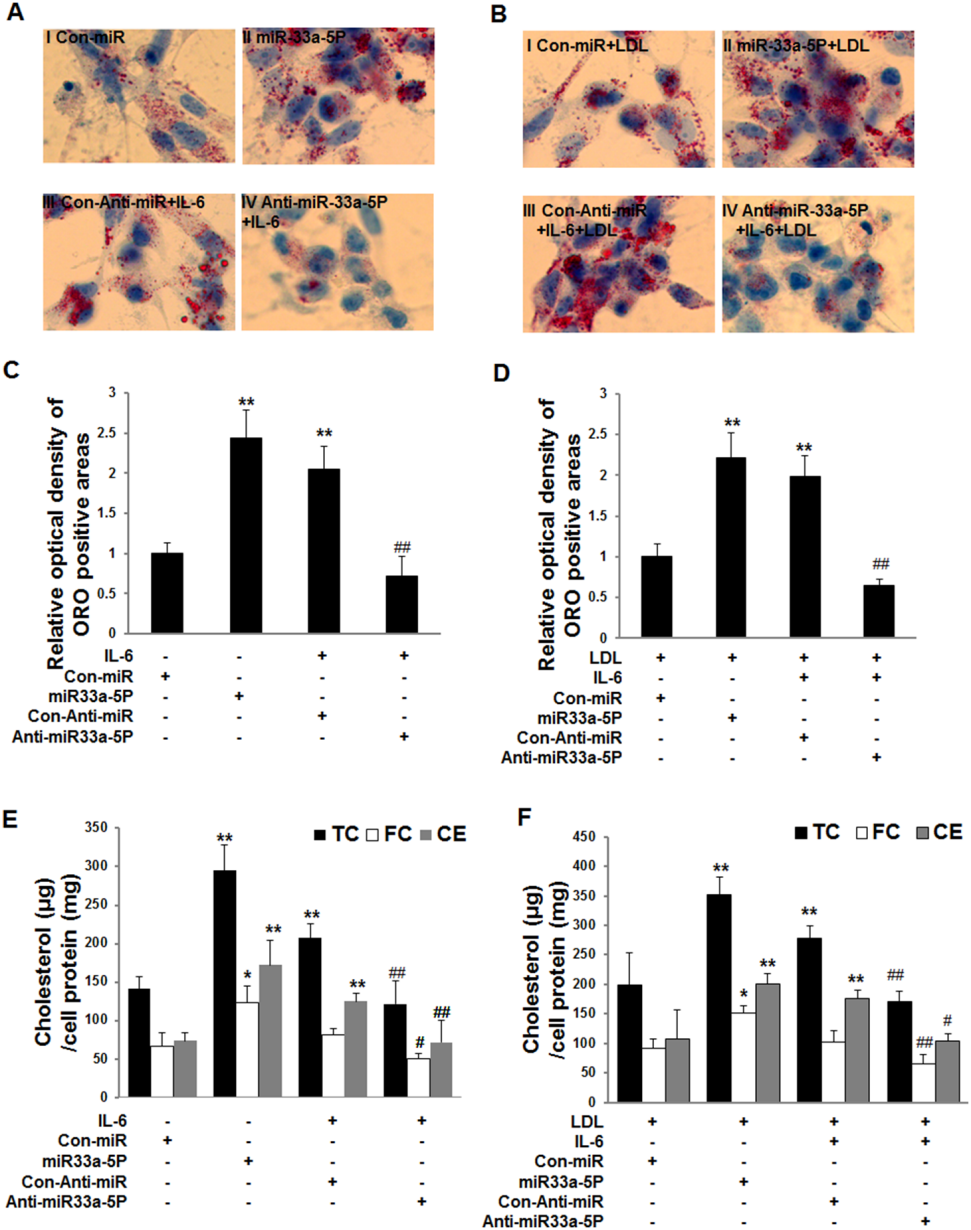
Effects of overexpression of miR-33a-5P and Anti-miR-33a-5P on intracellular lipid accumulation in THP-1 macrophages in the absence or presence of LDL. THP-1 macrophages were infected using Con-miR, miR-33a-5P, Con-Anti-miR, and Anti-miR-33a-5P, respectively, after 24 h PMA stimulation. After 48 h infection, THP-1 macrophages we incubated in serum-free medium at 37°C for 24 h. Then, the medium was respectively replaced by fresh serum-free medium (0.2% BSA) containing blank control (**A**, I, Con-miR), blank control (**A**, II, miR-33a-5P), 40 ng/ml IL-6 (**A**, III, Con- Anti-miR plus IL-6), 40 ng/ml IL-6 (**A**, IV, anti-miR-33a-5P plus IL-6), 25 µg/ml LDL (**B**, I, Con-miR plus LDL), 25 µg/ml LDL (**B**, II, miR-33a-5P plus LDL), 25 µg/ml LDL plus 40 ng/ml IL-6 (**B**, III, Con-Anti-miR plus IL-6 plus LDL), or 25 µg/ml LDL plus 40 ng/ml IL-6 (**B**, IV, Anti-miR-33a-5P plus IL-6 plus LDL), followed by incubation at 37°C for 24 h. (**A and B**) The cells were examined for lipid inclusions by oil red O staining. The results are representative of those observed in six separate experiments (×400). (**C and D**) Semi-quantitative analysis of oil red O positive staining. Data are means ± SD from 6 separate fields. (**E and F**) Quantification of levels of intracellular cholesterol contents. Data are means ± SD of duplicate wells from 6 experiments. *, P<0.05 compared with Con-miR or Con-miR plus LDL; **, P<0.01 compared with Con-miR plus LDL; #, P<0.05 compared with Con-Anti-miR plus IL-6 or Con-Anti-miR plus IL-6 plus LDL; ##, P<0.01 compared with Con- Anti-miR plus IL-6 or Con-Anti-miR plus IL-6 plus LDL.

Our data showed that intracellular lipid accumulation (Fig. 4A: II or III vs I; Fig. 4B: II or III vs I) and intracellular TC and CE (Fig. 4E and F) in both miR-33a-5P and IL-6 groups were increased in the absence or presence or LDL. However, overexpression of anti-miR33a-5P reduced lipid accumulation (Fig. 4A: VI vs III; Fig. 4B: VI vs III) and intracellular TC, CE and FC levels induced by IL-6 (Fig. 4E and F). Intracellular FC level was increased by overexpression of miR-33a-5P in comparison to Con-miR. However, IL-6 did not affect FC level in THP-1 macrophages (Fig. 4E and F).

### Effects of overexpression of miR-33a-5P and anti-miR-33a-5P on cholesterol efflux

**Figure 5:**
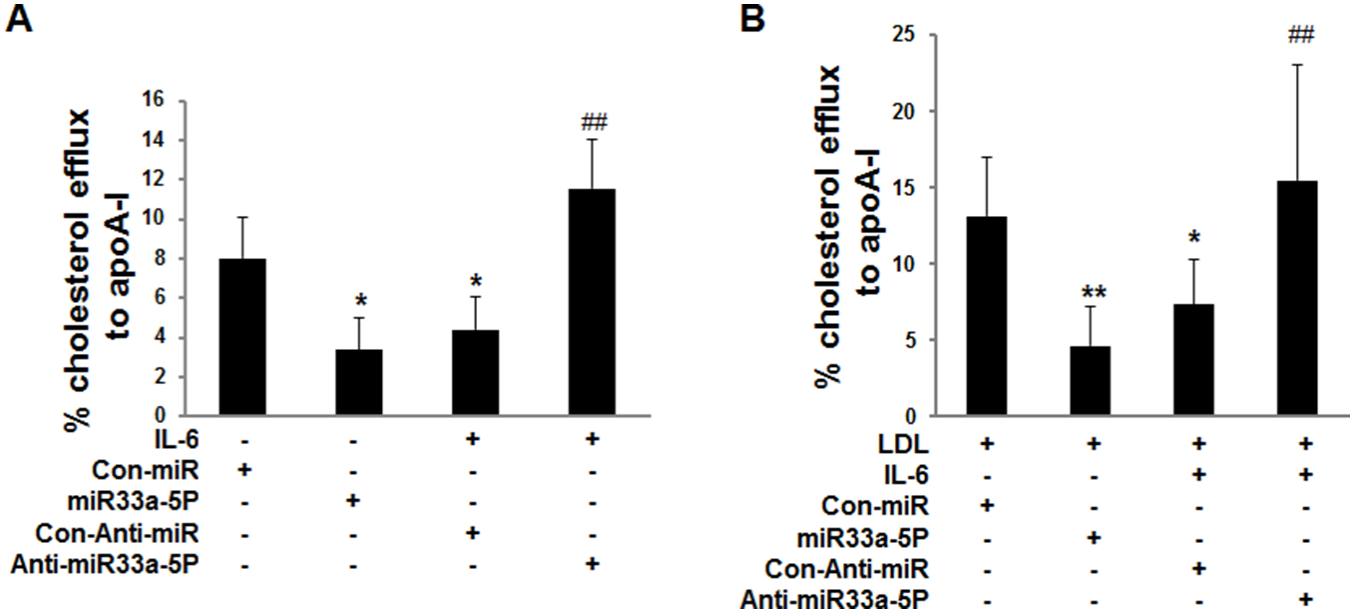
Effects of overexpression of miR-33a-5P and anti-miR-33a-5P on apoA-I mediated cholesterol efflux in THP-1 macrophages in the absence or presence of LDL. THP-1 macrophages were infected using Con-miR, miR-33a-5P, Con-Anti-miR, and anti-miR-33a-5P, respectively, after 24 h PMA stimulation. After 48 h infection, THP-1 macrophages were incubated in serum-free medium at 37°C for 24 h. The medium was then respectively replaced by fresh serum-free medium (0.2% BSA) containing (**A**) blank control, blank control, 40 ng/ml IL-6, or 40 ng/ml IL-6; (**B**) 25 µg/ml LDL, 25 µg/ml LDL, 25 µg/ml LDL plus 40 ng/ml IL-6, or 25 µg/ml LDL plus 40 ng/ml IL-6, followed by incubation at 37°C for 18 h. Next, the cells were cultured in serum-free medium containing treatment factors and 10 µg/ml apoA-I for 4 h. ApoA-I-mediated cholesterol efflux was assayed as described in the Materials and Methods section. Data are means ± SD of duplicate wells from 6 experiments. *, P<0.05 compared with Con-miR or Con-miR plus LDL; **, P<0.01 compared with Con-miR plus LDL; #, P<0.05 compared with Con-Anti-miR plus IL-6 or Con-Anti-miR plus IL-6 plus LDL; ##, P<0.01 compared with Con-Anti-miR plus IL-6 or Con-Anti-miR plus IL-6 plus LDL.

Our data showed that ApoA-I-mediated cholesterol efflux (Fig. 5A and B) was significantly decreased in THP-1 macrophages treated by miR-33a-5P and IL-6, in comparison to THP-1 macrophages infected with Con-miR in the absence or presence of LDL, suggesting that either inflammatory cytokines or miR-33a-5P increased intracellular lipid accumulation by decreasing apoA-I-mediated cholesterol efflux. By contrast, overexpression of anti-miR-33a-5P (Fig. 5A and B) reversed the effects of inflammatory cytokines in the absence or presence of LDL.

### Effects of overexpression of miR-33a-5P and anti-miR-33a-5P on the expression of miR-33a-5P, ABCA1 and ABCG1

**Figure 6:**
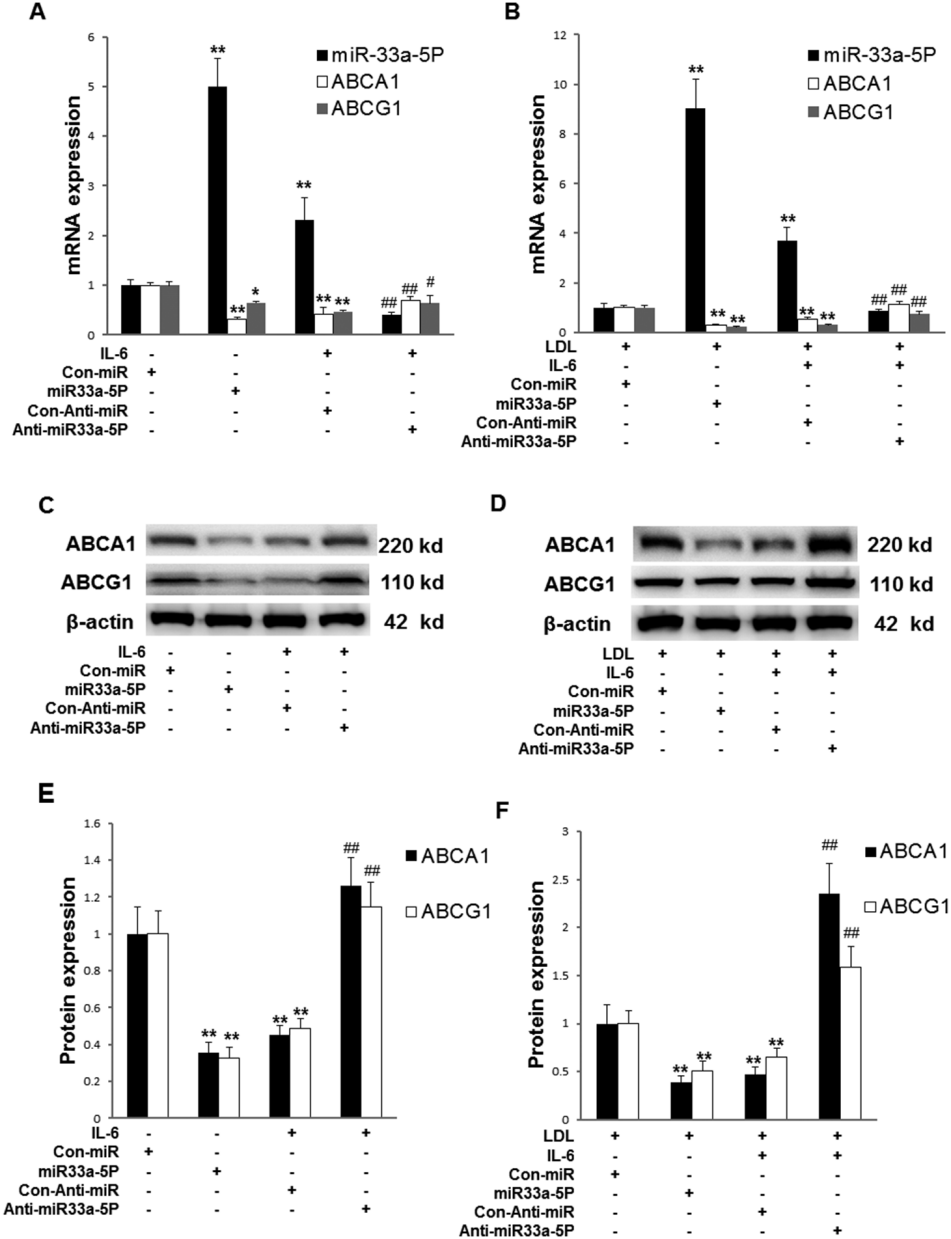
Effects of overexpression of miR-33a-5P and Anti-miR-33a-5P on the expression of miR-33a-5P, ABCA1 and ABCG1 in THP-1 macrophages in the absence or presence of LDL. THP-1 macrophages were infected using Con-miR, miR-33a-5P, Con-Anti-miR, and Anti-miR-33a-5P, respectively, after 24 h PMA stimulation. After 48 h infection, THP-1 macrophages were incubated in serum-free medium at 37°C for 24 h. The medium was then respectively replaced by fresh serum-free medium (0.2% BSA) containing (**A**) blank control, blank control, 40 ng/ml IL-6, or 40 ng/ml IL-6, (**B**) 25 µg/ml LDL, 25 µg/ml LDL, 25 µg/ml LDL plus 40 ng/ml IL-6, or 25 µg/ml LDL plus 40 ng/ml IL-6, followed by incubation at 37°C for 24 h. mRNA levels of miR-33a-5P, ABCA1 and ABCG1 were determined using RT-PCR. U6 or β-actin served as a reference gene. Data are means ± SD from 6 experiments. (**C and D**) Protein levels of ABCA1 and ABCG1 determined by Western blotting. (**E and F**) Quantification of densitometric values of ABCA1 and ABCG1 protein bands from four experiments, which were normalized to β-actin and expressed as a percentage of control. Data are means ± SD from 4 experiments. *, P<0.05 compared with Con-miR or Con-miR plus LDL; **, P<0.01 compared with Con-miR plus LDL; #, P<0.05 compared with Con-Anti-miR plus IL-6 or Con-Anti-miR plus IL-6 plus LDL; ##, P<0.01 compared with Con-Anti-miR plus IL-6 or Con-Anti-miR plus IL-6 plus LDL.

We showed that overexpression of miR-33a-5P and IL-6 stimulation increased miR-33a-5P expression that suppressed ABCA1 and ABCG1 mRNA and protein expression in THP-1 cells in the absence or presence of LDL (Fig. 6). However, overexpression of anti-miR-33a-5P overrode the inhibitory effect of IL-6 on ABCA1 and ABCG1 by reducing miR-33a-5P expression in the absence or presence of LDL (Fig. 6), suggesting that Anti-miR-33a-5P may reduce lipid accumulation by increasing ABCA1 and ABCG1 expression.

## Discussion

The excessive cholesterol accumulation and inflammation in vessel wall are two key factors for the development of atherosclerosis. Macrophages in the arterial wall derived from the circulatory monocytes are the main source of foam cells in atherosclerotic plaques [3]. The development of macrophage-derived foam cells that contain massive amounts of cholesterol esters is a hallmark of the early stage of atherosclerotic lesions [28], [29]. THP-1 macrophages are very similar to primary peripheral blood mononuclear cells in response to inflammatory cytokines [40], [41]. In the present study, we studied the role of inflammation on cholesterol efflux using THP-1-derived macrophages activated by PMA.

Our data demonstrated that inflammatory cytokines IL-6 or TNF-α, which were biomarkers of chronic systemic inflammation, promoted lipid accumulation in THP-1 macrophages. In addition to the results from oil red O staining in this study, the intracellular cholesterol quantitative measurements by enzymatic methods also showed that inflammatory cytokines increased TC and CE contents in THP-1 macrophages. Reverse cholesterol transport means that excessive cholesterol of peripheral tissue (e.g., blood vessels) is transported back to the liver for excretion in the form of bile after catabolism, and thus reduces excessive lipid accumulation under artery intima, preventing atherosclerosis [3]. Cholesterol efflux, which is mediated by ABCA1 and ABCG1, is the key step in reverse cholesterol transport. ABCA1 mediates the efflux of cholesterol from macrophages to apolipoprotein with poor lipid molecule like apolipoprotein A-I (apoA-I) [8], [10], [11]. The defects of ABCA1 gene leads to Tangier disease, which is characterized by extreme lack of HDL cholesterol in plasma, obstacles of cholesterol efflux and a great amount cholesterol deposition in peripheral tissue. Patients with Tangier disease have a higher risk for atherosclerosis [11]. ABCG1 mediates cholesterol efflux from macrophages to apolipoprotein with large lipid molecule like HDL [12]–[14]. In ABCG1 gene knockout mice, lipid metabolism is disordered, with a large amount of cholesterol being deposited in lung tissue and liver tissue [13]. miR-33 regulates the major risk factors of atherosclerosis, including lipid metabolism (cholesterol homeostasis, HDL biogenesis, phospholipid and triglyceride, fatty acid, and bile acid metabolism), inflammatory response, glucose/energy homeostasis and insulin signaling, cell cycle progression and proliferation, and myeloid cell differentiation [39]. Recent studies demonstrated that miR-33a is co-transcribed with SREBP-2 and binds to the 3′UTR of ABCA1/ABCG1 mRNA to inhibit ABCA1/ABCG1 translation and cholesterol efflux [20]–[24].

We previously demonstrated that inflammation increased lipid accumulation by increasing cholesterol uptake and synthesis [27]–[34]. In this study, we demonstrated that inflammatory cytokines decreased apoA-I-mediated cholesterol efflux. Furthermore, we demonstrated that inflammatory cytokines IL-6 or TNF-α enhanced the expression of miR-33a-5P and SREBP2, and decreased the expression of ABCA1 and ABCG1. Overexpression of miR-33a by inflammatory stress caused lipid accumulation by decreasing ABCA1 and ABCG1-mediated cholesterol efflux. However, anti-miR-33a-5P overrode the inhibitory effects of cholesterol efflux induced by inflammatory stress. These results suggested that anti-miR-33a-5P had beneficial effect on the prevention of lipid accumulation by increasing ABCA1 and ABCG1-mediated cholesterol efflux.

In summary, this study demonstrated that inflammatory cytokines significantly increased intracellular lipid accumulation by increasing miR-33a-5P expression that decreased ABCA1 and ABCG1-mediated cholesterol efflux. In addition, anti-miR-33a-5P reversed the effects of inflammatory cytokines on intracellular lipid accumulation and cholesterol efflux by overriding the role of miR-33a-5P in THP-1 macrophages. These results suggest that miR-33a-5P plays important roles in inflammatory responses and cholesterol efflux and that anti-miR-33a-5P may help prevent inflammatory cytokine-associated macrophage foam cell formation.
